# GPR88 promotes neurite outgrowth of sensory neurons via activation of G_i/o_

**DOI:** 10.3389/fphar.2025.1730247

**Published:** 2026-01-13

**Authors:** Didem Ün, Catherine Gilbert, Thomas Steinkellner, Isabella Salzer, Michael Freissmuth

**Affiliations:** 1 Institute of Pharmacology and the Gaston H. Glock Research Laboratories for Exploratory Drug Development, Centre of Physiology and Pharmacology, Medical University of Vienna, Vienna, Austria; 2 Department of Neuropharmacology and Neurophysiology, Centre of Physiology and Pharmacology, Medical University of Vienna, Vienna, Austria

**Keywords:** allosteric activators, dorsal root ganglia, GPR88, peripheral nerve injury, sensory neurons

## Abstract

**Introduction:**

The peripheral nervous system retains the intrinsic ability to regenerate: after nerve injury, axons can sprout and reinnervate their target organ. We hypothesized that this regenerative response was driven by a transcriptional program involving druggable gene targets that promote neurite outgrowth.

**Methods:**

Sensory neurons were isolated from rat dorsal root ganglia (DRG) and cultured in vitro. Transcript levels were determined by sequencing of RNA. RNAscope was used to visualize GPR88 transcripts in individual subtypes of DRG neurons. Neurite outgrowth was quantified in the absence and presence of allosteric activators of GPR88 to generate concentration-response curves. Neuronal cultures were incubated in the presence of pertussis toxin and gallein to block Gi/Go- and βγ-dependent signalling, respectively.

**Results & Discussion:**

Placing DRG neurons in culture severs their bipolar axons but they regrow spontaneously over several days. RNA sequencing revealed time-dependent differences in gene expression. The orphan G protein-coupled receptor GPR88 was robustly upregulated during the early phase of regeneration. A search in publicly available datasets confirmed that the mRNA encoding GPR88 was upregulated after peripheral nerve injury. RNAScope in situ hybridization visualized the expression of GPR88 in all major subtypes of DRG neurons. The allosteric activators of GPR88 (RTI-13951-33, racemic 2-PCCA and its enantiomers) promoted the neurite outgrowth in a concentration-dependent manner with EC_50_ values in the nanomolar range. Their effect was abolished by incubation with pertussis toxin and gallein. These observations identify GPR88 as a regulator of neurite outgrowth in DRG neurons and implicate Gi/Go as a component of the signaling pathway. The findings support the conclusion that GPR88 is a potential therapeutic target for accelerating peripheral nerve repair.

## 1 Introduction

Peripheral nerves can be severed due to accidental trauma or by both, elective and emergency surgical procedures. The incidence of peripheral nerve injury has been estimated to lie in the range of 7–14/100,000 (Asplund et al., 2009; Murphy et al., 2023). Peripheral nerves can regenerate. Surgical reconstruction exploits this regenerative capacity and is currently the only available treatment option. The outcome varies depending on many factors including the location and the nature of the injury, but in many instances the outcome is modest (Hundepool et al., 2015). Thus, long lasting or permanent functional impairment is seen in about 50% of the patients (Hundepool et al., 2015; Lee and Wolfe, 2000). The unmet medical need is evident.

Severing of peripheral nerves including the connective tissue creates distal and proximal axonal segments. The distal segments are subject to Wallerian degeneration, while the proximal axonal stumps first undergo retrograde degeneration before initiating regeneration by sprouting. Wallerian degeneration is accompanied by phenotypic changes in Schwann cells: their myelin production ceases and they transdifferentiate to a repair phenotype. Macrophages are recruited and activated to clear myelin debris. After the clearance of debris, Schwann cells align into longitudinal columns to form bands of Büngner that guide the growth of axonal sprouts toward their target tissues (Burnett and Zager, 2004; Liao et al., 2024). In spite of these intrinsic repair mechanisms, functional recovery is severely limited by the slow growth rate of axons, i.e., approximately 1–3 mm/day (Wujek and Lasek, 1983). Reinnervation is slower in people than in rodents, because a longer distance must be covered (Scheib and Höke, 2013). The slow rate of axonal regeneration accounts for the fact that reestablishing functional motor units or sensory connections can take several months or more than a year in clinical cases of traumatic peripheral nerve injury (Gordon, 2020). Failure to restore axonal connections in time can often lead to permanent functional impairment because of denervation atrophy (Gordon et al., 2011).

Axonotmesis, the severing of axons, triggers a cascade of adaptive changes within the neuron, which is driven by transcriptional reprogramming: accordingly, a large array of genes is differentially expressed in a time-dependent manner (Li et al., 2015; Renthal et al., 2020; Xu et al., 2022; Zhao et al., 2023). The focus of these previous analyses has been on understanding the hierarchy of transcription factors and their importance in supporting axon sprouting. In fact, the transcriptional response of aberrant regeneration differs from that required for successful reinnervation (Warner et al., 2023). Here, we searched for druggable targets by focusing on genes encoding G protein-coupled receptors (GPCRs) and solute carrier (SLC) transporters. Our approach relied on isolating neurons from rat dorsal root ganglia (DRG), which severed their axons. We surmised that the subsequent regeneration of neurites in cell culture was supported by a transcriptional program akin to that required for axonal sprouting *in vivo*. By leveraging transcriptomic profiling of DRG cultures over time, we identified the orphan GPCR GPR88 as a druggable candidate. We confirmed that GPR88 was expressed in all major subtypes of DRG neurons and that its stimulation by allosteric activators promoted neurite extension.

## 2 Materials and Methods

### 2.1 Reagents

Chemicals and reagents were: diethylpyrocarbonate (DEPC), dimethylsufoxide (DMSO), buffers and salts were purchased from Sigma-Aldrich (Vienna, Austria); (1R,2R)-N-[(2R,3R)-2-amino-3-methoxybutyl]-N-[4-[4-(methoxymethyl)phenyl]phe-nyl]-2-pyridin-2-ylcyclopropane-1-carboxamide (RTI-13951-33) hydrochloride (MedChemTronica, #HY-112612A), 2-pyridin-2-yl-cyclopropanecarboxylic acid (2-PCCA) hydro-chloride (MedChemTronica, #HY-100013C), (1R,2R)-2-PCCA hydrochloride (MedChemTronica, #HY-100013A1), (1S,2S)-2-PCCA hydrochloride (MedChemTronica, #HY-100013B1), S-(4-nitrobenzyl)-6-thioinosine (= nitrobenzylmercaptopurine ribonucleosi-de/NBMPR) (Sigma-Aldrich, #N2255), pertussis toxin (PTX) (Sigma-Aldrich, #P7208), gallein (MedChemExpress, #HY-D0254).

Cell culture media and constituents were: Dulbecco’s modified Eagle medium (DMEM) with high glucose (4.5 g/L), heat-inactivated fetal bovine serum (Biowest, Riverside, MO, United States); collagenase, penicillin, streptomycin, cytosine-β-D-arabinofuranoside hydrochloride (Ara-C), insulin, poly-D-lysine, 4′,6-diamidino-2-phenylindole (DAPI) (Sigma-Aldrich); nerve growth factor (Alomone Labs Ltd.); dispase II (Roche, Mannheim, Germany), trypsin (Worthington, Lakewood, NJ, United States).

Kits and reagents for molecular biology and RNAScope were: RNeasy Qiagen mini kit (Qiagen, Maryland, United States, #74104); NEB Next Poly(A) mRNA Magnetic Isolation Module and the NEB Next UltraExpress RNA Library Prep Kit (New England Biolabs); RNAscope Multiplex rat and RNAscope antisense probes against GPR88 (Rn-Gpr88, #445481) and RBFOX3 (Rn-RFOX3-C3, #436351-C3), NEFH (Rn-NEFH-C2, #474241-C2), P2RX33 (Rn-P2RX3-C3, #543401-C3), and CALCA (Rn-CALCA-C2, #317511-C2), Fluorescent Reagent Kit v2 (Advanced Cell Diagnostics/Bio-Techne Ireland Limited, Dublin, United Kingdom, #323290); Fluoromount-G mounting medium (Thermo Fisher Scientific).

### 2.2 Cell culture

Primary cultures of DRG neurons were prepared as previously described with slight modifications (Ray et al., 2019). Pregnant Sprague-Dawley rats were purchased from Charles River (Sulzfeld, Germany) and housed in a Scantainer (Scanbur, Karlslunde, Denmark) under a 12-h light-dark cycle, at 20 °C–25 °C, 40%–70% humidity, and food as well as water ad libidum. Rat pups of either sex aged 10–15 days were killed by decapitation in accordance with the ARRIVE guidelines (Percie du Sert et al., 2020), the guidelines of Good Scientific Practice by the Medical University of Vienna (https://www.ris.bka.gv.at/Dokumente/BgblAuth/BGBLA_2004_I_118/BGBLA_2004_I_118.html) and the Austrian animal protection law (https://www.ris.bka.gv.at/Dokumente/BgblAuth/BGBLA_2012_I_114/BGBLA_2012_I_114.html). The experiments were performed *ex vivo*. Wild-type animals were sacrificed, and their dorsal root ganglia were used subsequently. According to §1 of the Austrian animal experimentation law (https://www.ris.bka.gv.at/NormDokument.wxe?Abfrage=Bundesnormen&Gesetzesnummer=20008142&FassungVom=2025-09-07&Artikel=&Paragraf=1&Anlage=&Uebergangsrecht=), an approval of an animal ethics committee is not required for *ex vivo* experiments.

Ganglia were dissected from all levels of the spinal column and digested in collagenase (1.5 mg/mL) and dispase II (3.0 mg/mL) for 30 min, followed by trypsin (0.25%) for 10 min at 37 °C, respectively. Subsequently, the tissue was washed twice with Ca^2+^-free Tyrode solution (NaCl 150 mM, KCl 4 mM, MgCl_2_ 2 mM, glucose 10 mM, HEPES 10 mM, adjusted to pH 7.4 with NaOH) and triturated to produce a cell suspension in DMEM with high glucose (4.5 g/L) supplemented with 25,000 U/L penicillin, 25 mg/L streptomycin, 10 mg/L insulin, and 50 μg/L nerve growth factor. A total of 3,000 cells were seeded onto 35 mm culture dishes coated with poly-D-lysine. Cultures were maintained at 37 °C in a humidified 5% CO_2_ atmosphere and supplemented with 5% heat-inactivated fetal bovine serum 2 h after plating. The medium was exchanged on day 1 after seeding. Ara-C (100 µM) was added to inhibit glial cell proliferation.

### 2.3 RNA sequencing

DRG neurons were harvested immediately after dissection (time point 0 h), and after 2 h, 24 h (=day 1), 72 h (=day 3) and 168 h (=day 7) in culture to capture time-dependent changes. Total RNA was isolated using the RNeasy Qiagen mini kit according to the manufacturer’s protocol. Sequencing libraries from total RNA of the samples were prepared at the genomics core facility of the Medical University of Vienna, using the NEB Next Poly(A) mRNA Magnetic Isolation Module and the NEB Next UltraExpress RNA Library Prep Kit for Illumina with Unique Dual Indices (UDIs) according to the manufacturer’s protocols. Quality control of the libraries was done on a Bioanalyzer 2100 (Agilent) using a High Sensitivity DNA Kit for monitoring correct insert size and quantified using Qubit dsDNA HS Assay (Invitrogen). Libraries were sequenced on a P2 flow cell of a NextSeq2000 instrument (Illumina) in 1 × 76 bp single-end sequencing mode. Reads in fastq format were generated using the Illumina bcl2fastq command line tool (v2.19.1.403) including trimming of the sequencing adapters. On average, 30 million reads per sample were generated. Reads in fastq format were aligned to the rat reference genome version rnor 6.0 with Ensembl 96 annotations using STAR aligner (Dobin et al., 2013) version 2.6.1a in 2-pass mode. Raw reads per gene were counted by STAR. Differential gene expression was calculated using DESeq2 (Love et al., 2014) version 1.44.0 (Zhu et al., 2019). Transcripts per million (TPM) were quantified with the RSEM (RNA-Seq by Expectation Maximization) software (Li and Dewey, 2011). Volcano plots were generated with the web application VolcaNoseR, based on differentially expressed genes (DEGs) extracted from DESeq2 analysis (Goedhart and Luijsterburg, 2020).

### 2.4 Detection of GPR88 in rat dorsal root ganglia by fluorescent mRNA *in situ* hybridization

Rat DRG were excised and embedded *in toto* in Tissue-Tek® Optimal Cutting Temperature (O.C.T.) compound (Sakura Finetek purchased through Avantor - Austria VWR International GmbH, Vienna, Austria), frozen in liquid nitrogen, and stored at −80 °C until sectioning. Tissue sections (15 μm) were cut using a cryostat (CM3050S, Leica, Wetzlar, Germany) and mounted onto Superfrost Plus glass slides. Consecutive sections from each DRG were spaced at least 100 µm apart. The slides were stored at −80 °C until processing with the RNAscope Multiplex Fluorescent Reagent Kit v2. RNA detection was done with solutions provided in the kit according to the instructions of the manufacturer: sections were fixed in 4% paraformaldehyde (PFA) for 1 h at 4 °C, followed by two washes with phosphate-buffered saline and sequential dehydration in 50% ethanol for 5 min, 70% ethanol for 5 min, and 100% ethanol for 10 min at room temperature. The slides were then allowed to air-dry. Hydrophobic barriers were drawn around the tissue sections using a hydrophobic barrier pen. The sections were treated with hydrogen peroxide for 10 min, rinsed with sterile, diethylpyrocarbonate- (DEPC-) treated water and subsequently subjected to a protease IV digestion for 30 min at room temperature. After rinsing with DEPC-treated water, sections were covered with a solution containing rat RNAscope antisense probes against GPR88 and against the neuronal markers RBFOX3, NEFH, P2RX3 and CALCA in a 50:1 ratio (GPR88 probe to the other probes). The slides were placed into the HybEZ II oven (Advanced Cell Diagnostics) for 2 h at 40 °C. Subsequently, sections were rinsed twice in RNAscope wash buffer for 2 min and stored overnight in 5X SSC buffer (750 mM NaCl and 75 mM trisodium citrate, pH adjusted to 7.0 with HCl) at room temperature. On the next day, the slides were rinsed twice with RNAscope wash buffer, followed by amplification with Amp 1 for 30 min, Amp 2 for 30 min, and Amp 3 for 15 min at 40 °C, with additional rinses after each step. HRP-C1, HRP-C2, and HRP-C3 signals were developed by adding the corresponding RNAscope Multiplex FL v2 HRP reagents to the slides, followed by incubation in the HybEZ Oven at 40 °C for 15 min. Slides were then rinsed twice with wash buffer at room temperature. The sections were incubated with TSA Vivid Fluorophore 570 for C1-tagged probes and TSA Vivid Fluorophore 520 for C2- and C3-tagged probes for 30 min at 40 °C and then washed twice with wash buffer. Thereafter, a solution containing the HRP blocker was added and slides were incubated for 15 min at 40 °C before a final rinse with wash buffer. Nuclei were visualized by brief exposure to a solution containing 0.3 µM DAPI. The slides were then mounted with Fluoromount-G mounting medium, covered with a glass coverslip and stored at 4 °C. Images were captured by confocal laser scanning microscopy (Zeiss LSM 980 Airyscan 2) and processed using ImageJ. RNAscope signals were analysed qualitatively. According to criteria set forth by the manufacturer “A guide to RNAscope analysis” – http://fab.cba.mit.edu), cells are considered positive if they have ≥1 dot/cell. In our case, all neurons, which were scored positive had >2 dots/cell.

### 2.5 Neurite outgrowth

Twenty-four hours after seeding, neuronal cultures were incubated in the absence or presence of the indicated concentrations of RTI-13951-33 hydrochloride, 2-PCCA hydrochloride, 1R,2R-2-PCCA hydrochloride, 1S,2S-2-PCCA hydrochloride, or NBMPR for 24 h. In some instances, cultures were also incubated with 100 ng/mL PTX. Neurons and their neurite extensions were visualized by phase contrast microscopy (Nikon Eclipse TS100). Sholl analysis was performed by manually placing the neuronal soma into the center of the concentric circles, followed by measuring the distance (=radius) between the center and the most distant point reached by the longest neurite using the ImageJ (version 2.16.0) Neuroanatomy-plugin. This approach is conservative, because it underestimates total neurite length. However, it has the advantage to avoid ambiguities arising from tracing neurite extensions. Quantification was performed only on neurons, where neurite extensions were clearly identifiable. Neurons without neurite extension were not analyzed.

## 3 Results

### 3.1 Temporal evolution of transcript levels in cultured dorsal root ganglia neurons

**FIGURE 1:**
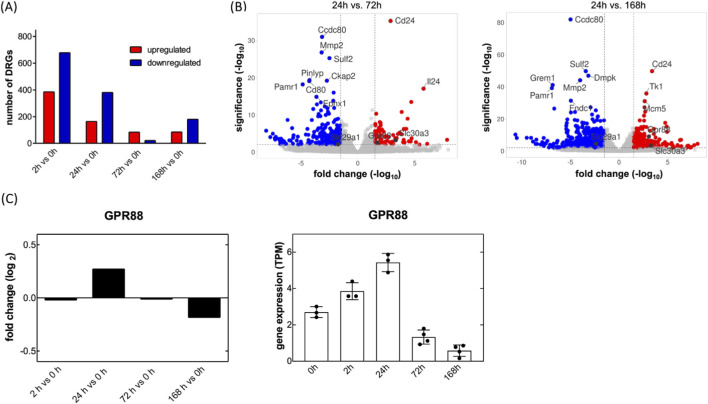
Transient up- and downregulation of transcripts encoding GPR88 and SLC29A1 in cultured rat dorsal root ganglia (DRG) neurons. **(A)** Primary neurons were isolated from dorsal root ganglia of rat pups and placed in culture. RNA was extracted immediately at the end of the isolation procedure (0 h) and after, 2, 24, 72 and 168 h in culture. Differential gene expression analysis was performed by comparing transcriptomic profiles in a time-dependent manner in three to four independent experiments. Differentially expressed gene transcripts (DEGS) were identified by DESeq2 differential gene expression analysis (Benjamini–Hochberg adjusted p-values of ≤0.05; fold change values ≥1). The numbers of upregulated genes are highlighted in red and the downregulated genes in blue. **(B)** The volcano plot shows gene expression profiles comparing day 1 to day 3 (left-hand panel) and day 7 (right-hand panel). The x-axis displays the log_2_ fold change in transcript levels, where positive values indicate upregulation and negative values indicate downregulation. The y-axis represents statistical significance, shown as the–log_10_ of the adjusted p-value. The dashed lines denote these threshold values. Genes that meet the thresholds of |log_2_ fold change| ≥ 1.5 and–log_10_ (p-adjusted) ≥ 2 are highlighted: red dots indicate significantly upregulated genes, blue dots indicate significantly downregulated genes, and grey dots represent genes without statistically significant changes. The annotated dots are the top ten genes with the greatest distance from the origin (calculated using the Manhattan distance) and the genes of interest, Gpr88 and SLC29A1. **(C)** Relative and absolute changes in transcripts encoding GPR88: transcript levels (transcripts per million = TPM) were determined at the indicated time points and are shown as log_2_ fold change vs. the 0 h time point (left hand plot) or as absolute values (right hand plot; dots represent individual experiments; bars and error bars correspond to means and S.D., respectively).

The peripheral nervous system has an inherent ability for self-repair, i.e., severed axons can sprout and re-innervate their target areas. Regenerating neurons activate a transcriptional program to support axon outgrowth. We surmised that this regenerative process was–at least in part–recapitulated *in vitro,* if sensory neurons were placed in culture: isolation of DRG neurons severs their axonal projections, but these begin to sprout again over a time course of several days. Accordingly, we monitored the evolution of transcriptional changes by RNA sequencing: RNA was isolated from freshly isolated DRG neurons (time point = 0 h) and 2, 24, 72, and 168 h after placing the neurons in culture. This time-resolved transcriptomic analysis aimed to capture the dynamic gene expression landscape underlying axonal regeneration. RNA sequencing revealed a total of 1,322 genes that were differentially expressed across all time points (DESeq2, adjusted p < 0.05) relative to the baseline (0 h). The most extensive transcriptional changes occurred after 2 h, with 386 genes upregulated and 678 downregulated, reflecting a rapid and robust early response to axonal injury (Figure 1A). This pattern reflects neuronal injury and the early phase of neuronal regeneration, which includes the rapid initiation of neurite outgrowth and gene activation. The gene expression remained significantly altered at 24 h (second set of bars in Figure 1A, 164 upregulated, 381 downregulated), suggesting continued transcriptional activity beyond the initial response. This stage likely represents the transition from the early to the late phase of regeneration. From 72 h onward, the number of DEGs declined markedly, consistent with a shift from an actively reprogramming to a more transcriptionally stable state that supports axonal elongation and maturation. We compared DEGs (DESeq2; adjusted p < 0.05, |log_2_ fold change| ≥ 1) between day 1 (24 h) at later time points, i.e., day 3 (72 h) and day 7 (168 h), to capture the transcriptional shift from early reprogramming to the more stable late phase of regeneration. This analysis identified 791 and 1,654 DEGs on day 1 relative to day 3 and day 7, respectively. Here, we focused on GPCRs and SLC transporters due to their relevance as druggable targets. One GPCR gene and 15 SLC genes were consistently differentially expressed in both comparisons (day 1 vs. day 3 and day 1 vs. day 7): in this relative comparison, transcripts encoding the GPCR GPR88 and SLC29A1, the equilibrative nucleoside transporter-1, were upregulated and downregulated, respectively (Figure 1B). SLC29A1 is druggable: in fact, the inhibitors of SLC29A1 dilazep and dipyridamole are approved drugs. Furthermore, NBMPR is an experimental inhibitor of SLC29A1, which has been used for some 5 decades as a research tool to interrogate the role of SLC29A1 (Pickard and Paterson, 1972; Paterson et al., 1980). We examined, if inhibition of SLC29A1 affected neurite outgrowth. The incubations were done in the presence of NBMPR rather than dipyridamole, because dipyridamole does not have any appreciable affinity for rat ENT1/SLC29A1, whereas the transporter is inhibited by NBMPR with an affinity in the range of 5 nM (Yao et al., 1997). However, incubation in the presence of 500 nM NBMPR, a quasi-saturating concentration (=100*K_D_), did not affect neurite outgrowth (Supplementary Figure S1). The other SLCs listed in Table 1 represent transporters, which translocate nutrients (glucose and amino acids), metabolites (monocarboxylates, citrate, uric acid), Zn^2+^, excitatory amino acids and choline. They are unlikely to represent suitable drug targets. Thus, we did not further pursue the solute carriers, but we focused on GPR88, which is also druggable: while the endogenous/orthosteric agonist of GPR88 is not known, GPR88 is activated by several synthetic compounds, which were reported to act as positive allosteric modulators, including 2-PCCA, RTI-13951-33 and RTI-122 (Jin et al., 2014; Jin et al., 2018; Chen et al., 2022; Rahman et al., 2023).

**TABLE 1:** Up- and downregulated transcripts encoding the indicated solute carriers (SLC) in cultured neurons isolated from rat dorsal root ganglia.^a^

Transcript	Full name and/or alias	Log2FoldChange day 1/Day 3	Log2FoldChange day 1/Day 7
SLC6A19	Sodium-dependent neutral amino acid transporter B0AT1	5.6569	5.1675
SLC2A2	Glucose transporter-2/GLUT2	5.9368	7.2332
SLC30A3	Zinc transporter-3/ZnT-3	3.3273	3.2609
SLC5A7	Choline transporter-1/CHT1	2.0943	2.0344
SLC37A1	Glucose 6-phosphate:inorganic phosphate antiporter/SPX1	1.5213	1.2218
SLC2A1	Glucose transporter-1/GLUT1	1.301	1.328
SLC16A1	Monocarboxylate transporter-1/MCT1	1.1002	1.2725
SLC38A3	Sodium-coupled neutral amino acid transporter-3/SNAT3	−3.2488	−1.6100
SLC2A12	Glucose transporter-12/GLUT12 (a urate transporter)	−3.1366	−3.448
SLC39A4	Zinc transporter ZIP4	−3.0438	−1.8118
SLC1A3	Excitatory amino acid transporter-1/EAAT1/GLAST1 (glial glutamate transporter)	−2.8961	−2.7675
SLC13A5	Sodium-citrate cotransporter/NACT	−2.5596	−2.1129
SLC39A8	Zinc transporter ZIP8	−1.9932	−1.9105
SLC29A1	Equilibrative nucleoside transporter/ENT1	−1.7380	−2.3576
SLC27A3	Long-chain fatty acid transport protein-3/FATP3	−1.6833	−1.3806

^a^ Transcript levels were quantified and compared as outlined in the legend to Figure 1.

The volcano plots shown in Figure 1B are based on a comparison of transcript levels in early and later stage DRG cultures. It is also instructive to examine the changes in transcript levels over the entire time course: transcripts for GPR88 rapidly reached a peak, i.e. 24 h after the DRG neurons had been placed into culture (Figure 1C).

**FIGURE 2:**
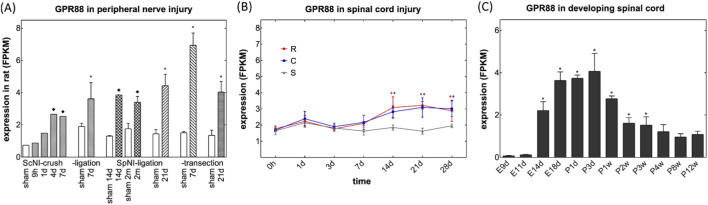
Mining of bulk RNA-sequencing data deposited in the DRGProfile database for changes in transcripts encoding GPR88. **(A)** Levels of transcripts encoding GPR88 **(A)** in FPKM (fragments per kilobase of exon per million mapped reads) after peripheral nerve injury. RNA was extracted from rat dorsal root ganglia, which were harvested at the indicated time points, i.e., after 9 h, 1 (1 d), 7 (7 d), 14 (14 d) and 21 days (21 d) or 2 months (2 m) from sham-treated animals (open bars) and after crushing (bars labeled ScNI_crush) or ligation of the sciatic nerve (bars labeled ScNI_ligation) and ligation (bars labeled SpNI_ligation) and transection of the spinal nerves (bars labeled SpNI_transection). Data were extracted from the deposition of Xu et al. (2022) and are means ± SD (n = 1-4); asterisks (*) indicates a statistically significant difference between injured and sham control groups with ≥3 samples (*p* < 0.05, one-tailed Welch’s t-test after Shapiro-Wilk test for normality); diamond (♦) indicates a significant difference between injured and sham control groups for n = 1 or n = 2 (log-transformed fold change >0.5 or < −0.5). **(B)** Levels of transcripts encoding GPR88in FPKM within the first 28 days after spinal cord injury in the segments rostral (red circles, R) and caudal (blue squares) to the lesion and in sham control animals (grey triangles, S). Data were extracted from the deposition by Yu et al. (2019) and represent means ± S.D. (n = 3). Changes in GPR88 transcripts differed in a statistically significant manner in rostral and caudal lesions on days 14–28; changes in SLC29A1 were significantly increased on days 7–21 and 14–21, respectively (*, p < 0.05; two-way ANOVA followed by Bonferroni comparison). **(C)** Levels of transcripts encoding GPR88 **(C)** in developing spinal cord. Time points of harvesting were embryonic days 9, 11, 14 and 18 (E9d, E11d, E14d, E18d), postpartum days 1 and 3 (P1d, P3d) and postpartum weeks 1–12 (P1w to P12w). Data were extracted from the deposition by Yang et al. (2021) and represent means ± S.D (n = 3). There was a statistically significant increase of GPR88 after E11d, which lasted to P3w (*, p < 0.05, ANOVA, followed by Bonferroni multiple comparison).

We verified our hits by mining the data compiled and deposited by Xu et al. (2022) in the DRGProfile database (http://121.41.67.1:3838/DRGProfile/) for time-dependent changes in transcript levels after peripheral nerve injury *in vivo*. Among the identified hits, GPR88 emerged as a particularly compelling candidate: GPR88 transcripts were consistently upregulated in rat DRG across various *in vivo* peripheral nerve injury models, i.e., crushing of (bars labeled ScNI_crush in Figure 2A) or ligation of the sciatic nerve (bars labeled ScNI_ligation in Figure 2A) and ligation (bars labeled SpNI_ligation, Figure 2A) and transection of the spinal nerves (bars labeled SpNI_transection, Figure 2A). In addition, induction of GPR88 appears to represent a general response to nerve injury: time-dependent accumulation of GPR88 transcripts can also be seen after spinal cord injury in the segments rostral and caudal to the lesion (Figure 2B; data extraction from the deposition by Yu et al., 2019). Finally, the transcriptomic data retrieved from the DRGProfile database (deposited by Yang et al., 2021) showed that *gpr88* was subject to pronounced developmental regulation*:* transcript abundance is low during the early embryonic stages, on embryonic days 9 and 11 (E9d and E11d in Figure 2C), but rises substantially on embryonic day 14 (E14d in Figure 2C), i.e., prior to the formation of synaptic contacts, which is initiated between embryonic days 15 and 17 (May and Biscoe, 1975). Transcript levels of GPR88 peak between embryonic day 18 and postnatal day 3 (E18d and P3d in Figure 2C) and subsequently decline to reach a steady state in postpartum weeks 8 and 12 (P8w and P12w in Figure 2C).

### 3.2 GPR88 expression across distinct DRG neuron subtypes

**FIGURE 3:**
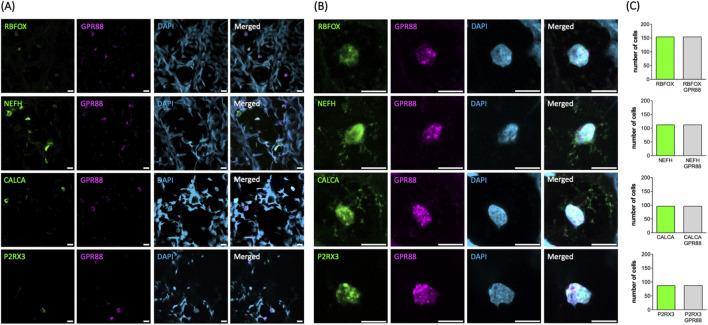
Detection of GPR88 by RNAScope analysis in whole DRG explants. **(A,B)** The markers for neuronal subtypes (RBFOX+, NEFH+, CALCA+, P2RX3+ in green, first column) and GPR88 (in magenta, second column) were visualized using the pertinent RNAScope probes. The cell nuclei were detected by DAPI staining (in blue, third column). Shown are representative images, which were captured by confocal microscopy (scale bar = 10 μm). Image overlays are shown in the right-hand panels. The overlay of the neuronal subtypes shown in green and GPR88 in magenta yields white as the indicator for co-localization. Panel **(B)** shows the magnified confocal images of representative neuronal nuclei from panel **(A)**. **(C)** The bar graphs summarize the distribution of all analyzed neurons according to marker expression. The y-axis represents the number of neurons expressing the indicated marker. All analyzed neurons (n = 449) showed expression of GPR88. Among them, 154, 112, 96 and 87 neurons were positive for RBFOX3, NEFH, CALCA and P2RX3, respectively.

DRG comprise functionally distinct neuronal subtypes specialized in transmitting different sensory modalities, including touch, temperature, proprioception, and pain. Transcriptomic profiling of single cells allowed for classifying these subtypes based on their characteristic gene expression patterns. Commonly defined groups include Aβ low-threshold mechanoreceptors (LTMRs), Aδ-LTMRs, C-LTMRs, peptidergic nociceptors (PEPs), non-peptidergic nociceptors (NPs), cold-sensitive neurons (TRPM8^+^), and proprioceptors (Usoskin et al., 2015; Wu et al., 2021). Among these, we selected the most prevalent subtypes to verify GPR88 expression across distinct DRG neuron populations using multiplex fluorescent mRNA *in situ* hybridization (RNAscope): the analysis confirmed robust GPR88 expression in all neurons, as identified by the marker RNA binding fox-1 homolog 3/neuronal nuclei (RBFOX3/NeuN; top row in Figures 3A,B). In line with this, GPR88 was consistently detected across distinct neuronal subtypes, including: (i) neurofilament heavy chain (NEFH) positive neurons, giving rise to large myelinated Aβ/Aα fibers involved in mechanoreception and proprioception (second row in Figures 3A,B), (ii) calcitonin gene-related peptide-α (CALCA) positive neurons, identifying small PEPs (giving rise to C-fibers) associated with pain and temperature sensing (third row in Figures 3A,B), and (iii) purinergic receptor 3 (P2RX3) positive neurons, labeling NPs (giving rise to C-fibers) involved in pain perception (bottom row in Figures 3A,B). Statistical analysis confirmed ubiquitous GPR88 expression in all neurons identified by the pertinent marker (Figure 3C). These results confirm that GPR88 is broadly expressed across major DRG neuronal subtypes. Conversely, it is evident from the images shown in Figure 3A that the non-neuronal cells, which were present in the slices prepared from the DRG, lacked any detectable reactivity for the GPR88 probe: their nuclei were visualized by DAPI (Figure 3A, third row) but were devoid of both, the fluorescence signal of the pertinent neuronal marker and of GPR88 (cf. merged images in the fourth row of Figure 3A).

### 3.3 Enhanced neurite outgrowth in early postnatal DRG neurons in response to GPR88 activators

**FIGURE 4:**
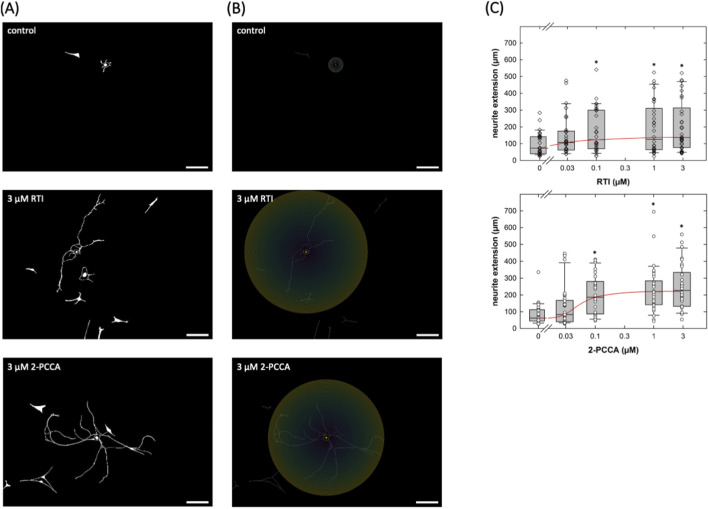
Neurite outgrowth in cultured DRG neurons incubated in the presence of the GPR88 agonists. **(A,B)** DRG neurons were isolated and seeded on day 0; after 24 h vehicle or compounds were added to reach the indicated final concentrations of RTI-13951-33 (RTI) or of (racemic) 2-PPCA. The incubation lasted for 24 h, i.e., images were captured by phase contrast microscopy on day 2. Shown are representative micrographs of primary DRG neurons (nominal microscopic magnification 100x): images were inverted in ImageJ (scale bar = 100 µm). **(B)** illustrates the principle of the Sholl analysis which was used to define neurite extension. The Neuroanatomy-plugin in ImageJ (version 2.16.0) was used to measure the radius as the distance from the soma to the farthest extent of the longest neurite. **(C)** Concentration-response curves were generated with the indicated concentrations of RTI-13951-33 (RTI, upper panel) or of 2-PPCA (lower panel). Neurite outgrowth was quantified by measuring the distance between the soma and the most distant point reached by the longest neurite as outlined under Materials and Methods (n = 3 independent experiments with a total 30 neurons/data point). Boxes show the median and the interquartile range, whiskers represent 95% confidence interval. Symbols represent individual determinations. Neurite extension differed significantly from that seen in control cultures at concentrations ≥0.1 µM (* vs. control, p < 0.05; Kruskal–Wallis test followed by *post hoc* testing using the Student-Newman-Keuls method).

Several compounds, including 2-PCCA and its congeners, were identified as synthetic agonists of GPR88 some 10 years ago (Jin et al., 2014). RTI-13951-33 is an analogue of 2-PCCA with improved selectivity and pharmacokinetics (Jin et al., 2018). The structure of GPR88 was elucidated at atomic resolution by cryo-electron microscopy and revealed 2-PCCA in an allosteric site rather than the canonical orthosteric ligand binding pocket (Chen et al., 2022). Accordingly, we tested whether these allosteric activators enhanced neurite outgrowth in cultured DRG neurons. Cultures of DRG neurons were treated with increasing concentrations of RTI-13951-33 or 2-PCCA for 24 h; thereafter microscopic images were captured: representative micrographs obtained from cultures incubated in the absence (Figure 4A top) and presence of a quasi-saturating concentration (3 µM) of RTI-13951-33 (Figure 4A middle) or of 2-PCCA (Figure 4A bottom) illustrate that incubation of DRG neurons with either compound for 24 h promoted neurite outgrowth. We generated concentration-response curves by quantifying the distance covered by the longest neurite in randomly selected neurons: incubation of DRG cultures in the presence of both, RTI-13951-33 (Figure 4B, top panel) and 2-PCCA (Figure 4B, middle panel) enhanced neurite growth in a concentration-dependent manner. A statistically significant increase in neurite extension was observed at concentrations ≥0.1 µM (Kruskal–Wallis test followed by Dunn’s *post hoc* test). From the concentration-response curve, we calculated EC_50_ values of 22.3 ± 9.8 nM and 67.8 ± 44.8 nM for RTI-13951-33 and 2-PCCA, respectively.

**FIGURE 5:**
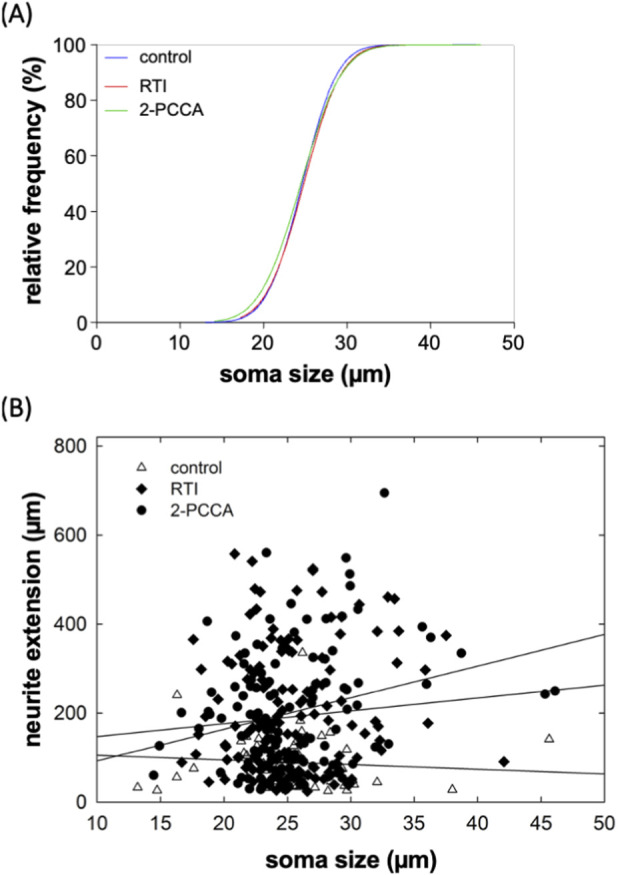
Distribution of soma size of DRG neurons **(A)** and lack of correlation between soma size and neurite extension **(B)**. **(A)** The soma size (largest diameter) of each neuron analyzed in the panels of Figure 4A was measured. The plot shows the cumulative distributions of the soma sizes of the neurons incubated in the absence (control, blue curve) and presence of RTI-13951-33 (RTI, red curve) or of 2-PPCA (green curve). The distribution is shown as percentage, because the total numbers of neurons differed: n = 60 for control and 120 each for RTI-13951-33 and 2-PPCA. **(B)** Scatter plot showing the neurite extension as a function of soma size for the control neurons (open triangles), and neurons treated with 1 µM RTI-13951-33 (closed diamonds) and 1 µM 2-PPCA (closed circles). The lines were drawn by linear regression: *R* ^2^ was 0.0066, 0.0071, and 0.0673, for control, RTI-13951-33 and 2-PPCA, respectively indicating that there was not any significant correlation between neurite length and soma size.

Our assessment was based on randomly selecting neurons for measuring neurite outgrowth. We verified that there was no selection bias by comparing the distribution of soma sizes of the neurons selected for assessing the action of RTI-13951-33 and of 2-PCCA with those selected from the corresponding control cultures: the cumulative distributions were comparable (Figure 5A). We also plotted the neurite extension of neurons in control cultures and in cultures treated with RTI-13951-33 and of 2-PCCA (each at 1 µM) as a function of soma size and examined, if there was any correlation (Figure 5B). This analysis showed that neurite length was independent of soma size (*R* ^2^ = 0.0066, 0.0071 and 0.0673 for neurons incubated under control, in the presence of RTI-13951-33 and of 2-PCCA, respectively). Thus, consistent with the RNAscope results, which indicated that all major subtypes of neurons expressed GPR88, the promotion of neurite outgrowth by RTI-13951-33 and of 2-PCCA was not confined to a subpopulation on neurons.

**FIGURE 6:**
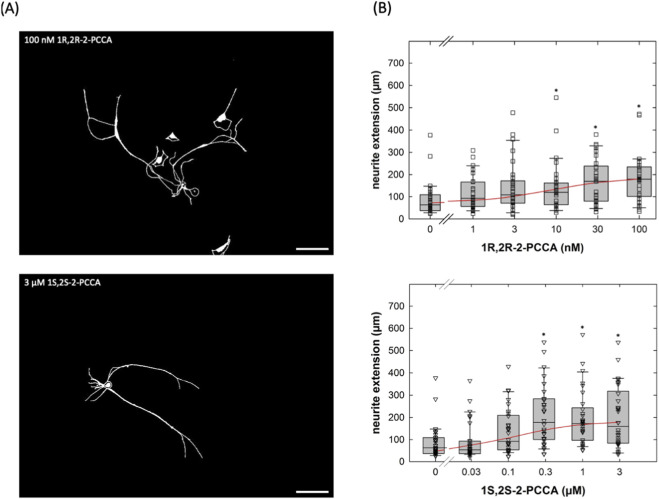
Concentration-response curves for the enantiomers 1R,2R-2-PCCA and 1S,2S-2-PCCA in promoting neurite outgrowth of cultured DRG neurons. **(A)** DRG neurons were incubated in the presence of 100 nM 1R,2R-2-PCCA (top panel) and of 3 µM 1S,2S-2-PCCA (bottom panel) as outlined in the legend to Figure 4A. After 24 h, images were captured by phase contrast microscopy and inverted using ImageJ (scale bar = 100 µm). **(B)** Concentration-response curves were generated with the indicated concentrations of 1R,2R-2-PCCA (top panel) and 1S,2S-2-PCCA (bottom panel). Neurite outgrowth was quantified by measuring the distance between the soma and the most distant point reached by the longest neurite as outlined under Materials and Methods (n = 3 independent experiments with a total 30 neurons/data point). Boxes show the median and the interquartile range, whiskers represent 95% confidence interval. Symbols represent individual determinations. Neurite extension differed significantly from that seen in control cultures at concentrations ≥10 nM 1R,2R-2-PCCA and ≥0.3 µM 1S,2S-2-PCCA (* vs. control, p < 0.05; Kruskal–Wallis test followed by *post hoc* testing using the Student-Newman-Keuls method). Experiments with the two enantiomers were done in parallel. Accordingly, the control incubations in the absence of compounds show the same values.

2-PCCA is a racemic mixture consisting of the 1R,2R- and 1S,2S-enantiomers. The 1R,2R-enantiomer was previously shown to be approximately 5-fold more potent in activating GPR88 than the 1S,2S-isomer (Jin et al., 2014). Accordingly, we capitalized on the availability of the enantiomers to further confirm that the neurite outgrowth promoting action of 2-PCCA and its analogue RTI-13951-33 were due to engaging GPR88 rather than to an off-target effect. 1R,2R-2PCCA was substantially more potent than 1S,2S-2PCCA (Figures 6A,B): the EC_50_-values estimated from the concentration-response curves were 9.1 ± 5.0 nM and 138.3 ± 98.4 nM for the 1R,2R- and the 1S,2S-enantiomer, respectively.

**FIGURE 7:**
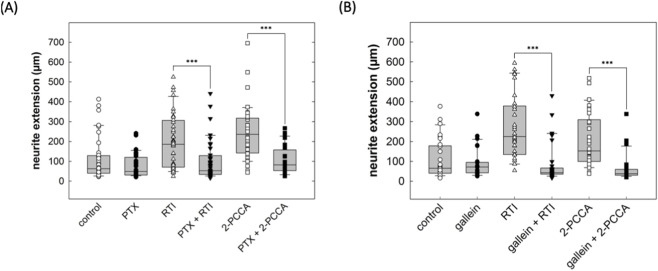
Inhibition by pertussis toxin and gallein of the neurite growth promoting effect of GPR88 agonists in DRG neurons. **(A)** DRG neurons were incubated in the absence and presence of pertussis toxin (PTX, 100 ng/mL) alone or in combination with the GPR88 agonists 2-PCCA (1 µM) and RTI-13951-33 (1 µM). After 24 h, neurite outgrowth was assessed by phase contrast microscopy and quantified as outlined in the legend to Figure 4. Boxes show the median and the interquartile range from a total of 40 neurons from 4 independent experiments; whiskers represent 95% confidence interval. Symbols represent individual determinations. In the absence of GPR88 agonists, PTX did not affect neurite extension compared to vehicle control (p = 0.20). However, in DRG cultures treated with the GPR88 agonists RTI-13951-33 and 2-PCCA, neurite extension differed in a statistically significant manner in the absence and presence of PTX (***, p < 0.001, Mann-Whitney U-test). **(B)** Neurite outgrowth in DRG neurons in the presence of the Gβγ inhibitor gallein (10 µM) alone or in combination with the GPR88 agonists 2-PCCA (1 µM) and RTI-13951-33 (1 µM) was quantified as described for **(A)**. In the absence of GPR88 agonists, gallein reduced neurite extension compared to control without reaching statistical significance. In cultures incubated in the presence of 2-PCCA or RTI-13951-33, neurite extension was significantly reduced in the presence of gallein (***, p < 0.001, Mann-Whitney U-test).

Upon activation, GPR88 preferentially recruits G_i/o_ family members (Jin et al., 2014; Cheng et al., 2022; Watkins and Orlandi, 2021), coupling to other classes of heterotrimeric G proteins (G_s_, G_q_, G_12/13_) was not detectable (Watkins and Orlandi, 2021). Thus, the action of the allosteric activators 2-PCCA and its analogue RTI-13951-33 are predicted to be blocked by pertussis toxin. This prediction was verified: if cultured DRG neurons were treated with pertussis toxin (100 ng/mL), 2-PCCA (1 µM) and RTI-13951-33 (1 µM) failed to promote neurite outgrowth (right-hand set of bars in Figure 7A). Importantly, PTX did not affect the neurite length in control cultures incubated in the absence of GPR88 allosteric agonists (cf. first two bars in Figure 7). We further probed the signaling pathway by exploring the effect of gallein, a small molecule high-affinity inhibitor of Gβγ-dependent signaling (Lehmann et al., 2008; Seneviratne et al., 2011): incubation in the presence of gallein (10 µM) did not affect neurite outgrowth under control conditions (cf. first two bars in Figure 7B), but it abolished the stimulation of neurite outgrowth by both, 2-PCCA (1 µM) and RTI-13951-33 (1 µM).

## 4 Discussion

GPR88 was originally identified as STRG, the striatum-specific GPCR (Mizushima et al., 2000). Accordingly, GPR88 has been explored as a candidate drug target in alcohol addiction (Jin et al., 2018; Ben Hamida et al., 2022; Anversa et al., 2024; Lovelock et al., 2025), schizophrenia (Lu et al., 2024) and Parkinson’s disease (Mantas et al., 2020). However, expression of GPR88 in the forebrain is not confined to the striatum but is also seen e.g., in the cerebral cortex and the amygdala (Massart et al., 2016). In fact, offsprings of consanguineous parents, who harbor a biallelic disruption of *gpr88*, develop a syndrome, where learning disabilities and delayed speech acquisition precedes frank choreatic movement (COCPMR = childhood-onset chorea with psychomotor retardation; Alkufri et al., 2016). The phenotype of mice with global disruption of *gpr88* is also more complex (Logue et al., 2009; Quintana et al., 2012; Meirsman et al., 2016a) than the one with targeted deletion in striatal medium-size spiny neurons of the indirect pathway (Meirsman et al., 2016b). Here, we show that expression of GPR88 was induced by severing the axons of DRG neurons and that activation of GPR88 promoted neurite outgrowth in DRG neurons. Our conclusions are based on the following sets of observation: (i) An induction of *gpr88* was seen after both, placing DRG neurons in culture and peripheral nerve injury. (ii) GPR88 transcripts were consistently visualized in all major subtypes of neurons in slices prepared from excised DRG, but they were absent in non-neuronal cells. Thus, while our transcriptomic analysis inevitably also covered mRNA from other cell types, which were present in the culture dishes (Schwann cells, fibroblasts etc.) and this is also true for the analysis of peripheral nerve injury models, we do not consider this limitation a source of concern, because GPR88 transcripts were confined to DRG neurons. (iii) The two allosteric agonists 2-PCCA and RTI-13951-33 promoted neurite outgrowth with EC_50_ values in the nanomolar range and with the potency of RTI-13951-33 exceeding that of (racemic) 2-PCCA, which is consistent with an action on GPR88 (Jin et al., 2014; Jin et al., 2018). 2-PCCA and RTI-13951-33 are hydrophobic lipid-like molecules and thus prone to give rise to non-specific effects. In fact, lipoamines can directly stimulate nucleotide exchange by heterotrimeric G proteins (Breitweg-Lehmann et al., 2002). At micromolar concentrations (≥3 µM), 2-PCCA also stimulates binding of GTPγS to membranes, which are devoid of GPR88 (Jin et al., 2018). However, an off-target effect of 2-PPCA can be ruled out based on the pronounced stereoselectivity, which was observed: as expected for an action via GPR88, 1R,2R-2PCCA was more potent than the 1S,2S-enantiomer and racemic 2-PCCA. (iii) Finally, pertussis toxin and gallein reversed the action on neurite outgrowth of the allosteric agonists. Taken together, these findings provide compelling evidence for a role of GPR88 in supporting neurite outgrowth of DRG neurons.

Neurite outgrowth is contingent on microtubule assembly (Drubin et al., 1985). The dynamics of tubulin assembly and disassembly is subject to bidirectional regulation by Gα- and Gβγ-subunits (Roychowdhury and Rasenick, 2008), where Gα_i-1_ and Gα_o_ activate the GTPase of β-tubulin (Roychowdhury et al., 1999) and Gβγ-subunits promote tubulin polymerization (Roychowdhury and Rasenick, 1997). Signaling by GPCRs is mediated by both, the Gα- and the Gβγ-subunits. In neurons, members of G_i_/G_o_-subfamily, in particular G_o_, are the most abundant heterotrimeric G proteins. Because of their abundance, they serve as a source of Gβγ-subunits. By ADP-ribosylating the cysteine residue four amino acids removed from the C-terminus, pertussis toxin abrogates binding of receptors to Gα_i_/Gα_o_-subunit, the subsequent guanine nucleotide exchange and dissociation of Gα and Gβγ (Freissmuth et al., 1989). Thus, after exposure of DRG neurons to pertussis toxin, activation of GPR88 failed to release free Gβγ and this *per se* suffices to explain the inhibition of neurite outgrowth (Sierra-Fonseca et al., 2014). We verified the role of Gβγ-subunits by employing gallein, a small-molecule inhibitor, which selectively blocks signaling by released Gβγ-subunit with submicromlar affinity (Lehmann et al., 2008; Seneviratne et al., 2011). Incubation of DRG neurons with gallein abolished the neurite outgrowth-promoting effects of GPR88 agonists, consistent with a requirement for Gβγ-mediated signaling. Regulation of tubulin elongation by Gβγ-subunits is the obvious candidate mechanism (Sierra-Fonseca et al., 2021), but additional signaling pathways may contribute to the action of GPR88 (Bromberg et al., 2008).

Of the 149 GPCR transcripts, which were detected in our transcriptomic analysis, GPR88 was the only one, which was subject to a statistically significant upregulation over the time course studied. We note that there was a discrepancy between the transient, rapid rise in GPR88 transcripts in cultured DRG neurons and the more sustained expression, which was seen in the *in vivo* models of peripheral nerve injury. This discrepancy presumably arises from the sustained regenerative response, which occurs during axonal sprouting *in vivo*.

It is a truism that protein levels are not strictly correlated with mRNA levels, i.e., it is not possible to infer how many molecules of a given protein are made from an mRNA copy. However, the abundance of a given protein at steady-state is primarily determined by the mRNA level (Liu et al., 2016). A perturbation, which leads to an up- or downregulation of the mRNA, can lead to a poor correlation between mRNA levels and protein abundance, until the new steady-state is reached (Liu et al., 2016). Because the relation between mRNA and GPR88 is not known for GPR88, it is not clear when receptor expression will peak and how rapidly it will decline. This limitation, however, does not detract from the fact that activation of GPR88 did promote neurite outgrowth.

A recent report also documented that GPR88 transcripts were elevated in DRG neurons of rats, which had been administered paclitaxel (Sankaranarayanan et al., 2025). Taxanes disrupt the regulation microtubular dynamics and thus cause axonal damage. Hence, it is attractive to posit that induction of *gpr88* is a general response of DRG neurons to neuronal damage. Axotomy induces the expression of the transcription factor ATF3 in DRG neurons; ATF3 is one of the master regulators, which orchestrate the response of DRG neurons to axotomy (Renthal et al., 2020). Administration of paclitaxel also triggers ATF3 expression in DRG neurons (Peters et al., 2007). However, we failed to find any canonical ATF3 binding sites within the candidate promoter region (i.e., up to 5,000 bp upstream of the start codon) of *gpr88*. Thus, it is at present not possible to infer, how rapid regulation of GPR88 transcripts is brought about.

The limitation of our study is that it was conducted *in vitro* and only focused on accelerating neurite extension of DRG neurons. *In vivo*, regeneration after traumatic peripheral nerve injury is contingent on a concerted response of several cell types (Gordon, 2020). Nevertheless, axonal outgrowth is a fundamental prerequisite for peripheral nerve regeneration: it is clear that nerve regeneration cannot be accomplished *in vivo*, if axons fail to sprout. In fact, the slow intrinsic elongation rate is a rate limiting factor (Höke and Brushart, 2010).

It is also important to consider that most publicly available DRG datasets are generated from adult tissue, while our study relies on DRG prepared from neonatal rat pups. Notably, gene expression profiles and cellular composition of the tissues changes with age: Adult DRGs contain a greater number of neurons and a different proportion of non-neuronal cells, such as satellite glial cells and Schwann cells, which can affect transcriptomic profiles and functional outcomes (Popken and Farel, 1997; Zhuo et al., 2025). Although neurite outgrowth is known to vary with developmental stage, previous studies show that DRG neurons of all ages retain substantial regenerative potential when inhibitory myelin-derived cues are removed, suggesting that age-related differences do not fully preclude comparable injury responses (Ng and Lozano, 1999). Age-dependent differences in neuronal maturation, cellular composition, and transcriptional profiles may therefore influence the extent to which our findings align with *in vivo* datasets, and this discrepancy should be taken into account when comparing across studies. Yet, the fact that GPR88 and SLC29A1 were upregulated after nerve injury in both our neonatal and adult rats from other studies supports the robustness and importance of these genes.

Currently, there is not any treatment, which has been approved for promoting regeneration of peripheral nerves, although many approaches are being studied (Modrak et al., 2020). Our findings justify pursuing GPR88 agonists as a viable option for the treatment of peripheral nerve injury. At the very least, it can be argued that the compounds, which we tested have shown activity when tested in animal models (Jin et al., 2018; Ben Hamida et al., 2022).

## Data Availability

The RNA sequencing data were submitted to the NCBI Sequence Read Archive and are available under the accession number PRJNA1375777. The measurements of neurite extension, which are the basis for the concentration-response curves and the box plots in Figures 4, 6, 7 are compiled in tabulated from in Supplementary Material-1. Further inquiries can be directed to the corresponding author.
