# Supplementary material for: GPR88 promotes neurite outgrowth of sensory neurons via activation of Gi/o
Source: Front Pharmacol. 2026 Jan 13;16:1730247. doi: 10.3389/fphar.2025.1730247 (PMC12835199; doi:10.3389/fphar.2025.1730247)
Supplement: Supplementary file 1 [file Supplementaryfile1.zip › Figure S1.PDF]

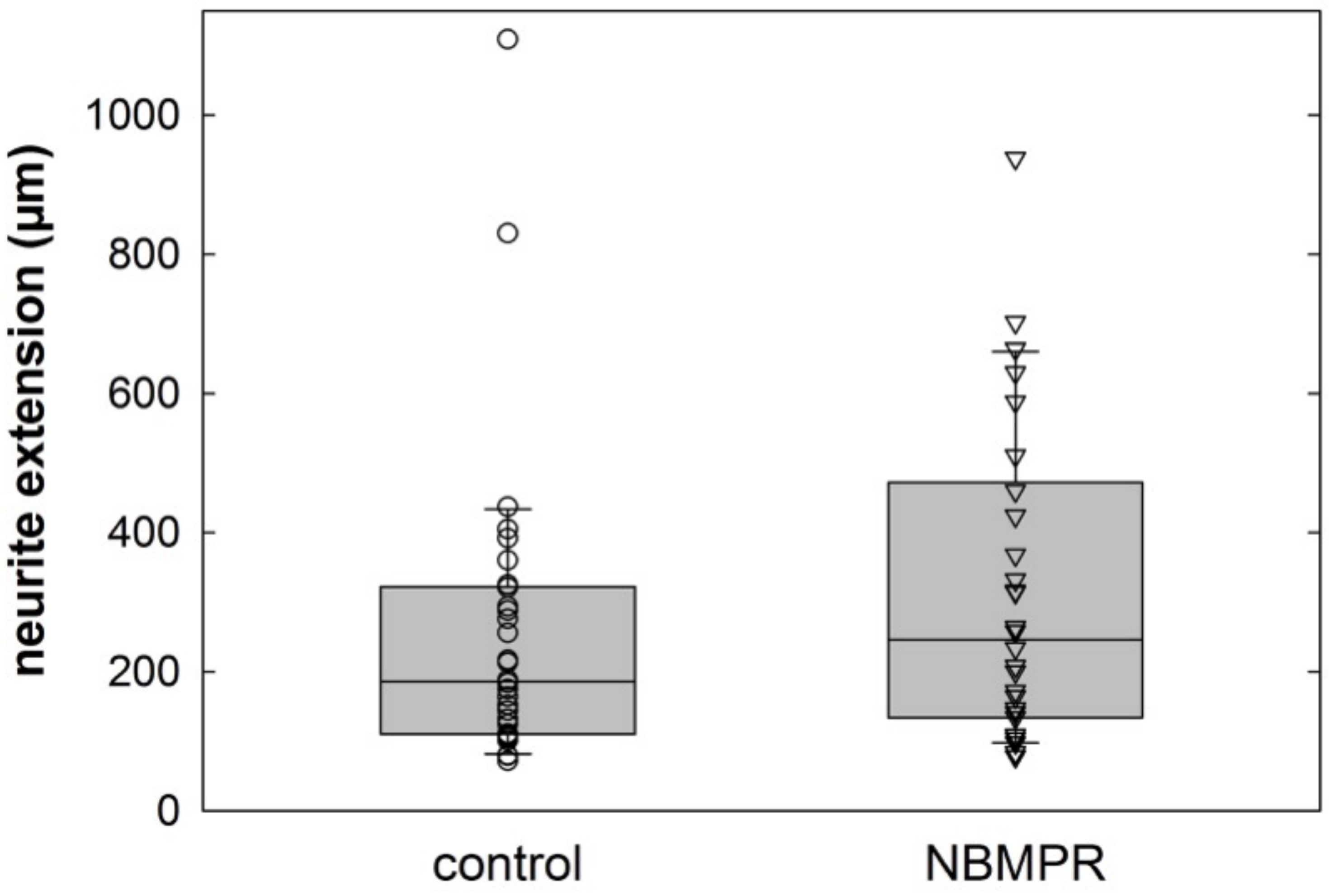

Fig. S1. Neurite outgrowth in cultured DRG neurons incubated in the presence of the SLC29A1 inhibitor NMPR. DRG neurons were isolated and seeded on day 0; after 24 h vehicle or NMR (0.5 µM final concentration) were added. The incubation lasted for 24 h, i.e. images were captured by phase contrast microscopy on day 2. Neurite outgrowth was quantified by measuring the distance between the soma and the most distant point reached by the longest neurite as outlined under Materials and Methods and in the legend to Fig. 4 (n = 3 independent experiments with a total 30 neurons/data point). Boxes show the median and the interquartile range, whiskers represent 95% confidence interval. Symbols represent individual determinations. NMPR did not affect neurite outgrowth in a statistically significant manner (p= 0.3, Mann-Whitney U-test)
