# Supplementary material for: GPR88 promotes neurite outgrowth of sensory neurons via activation of Gi/o
Source: Front Pharmacol. 2026 Jan 13;16:1730247. doi: 10.3389/fphar.2025.1730247 (PMC12835199; doi:10.3389/fphar.2025.1730247)
Supplement: Supplementary file 1 [file Supplementaryfile1.zip › Figures S2-S5.PDF]

Figure S2

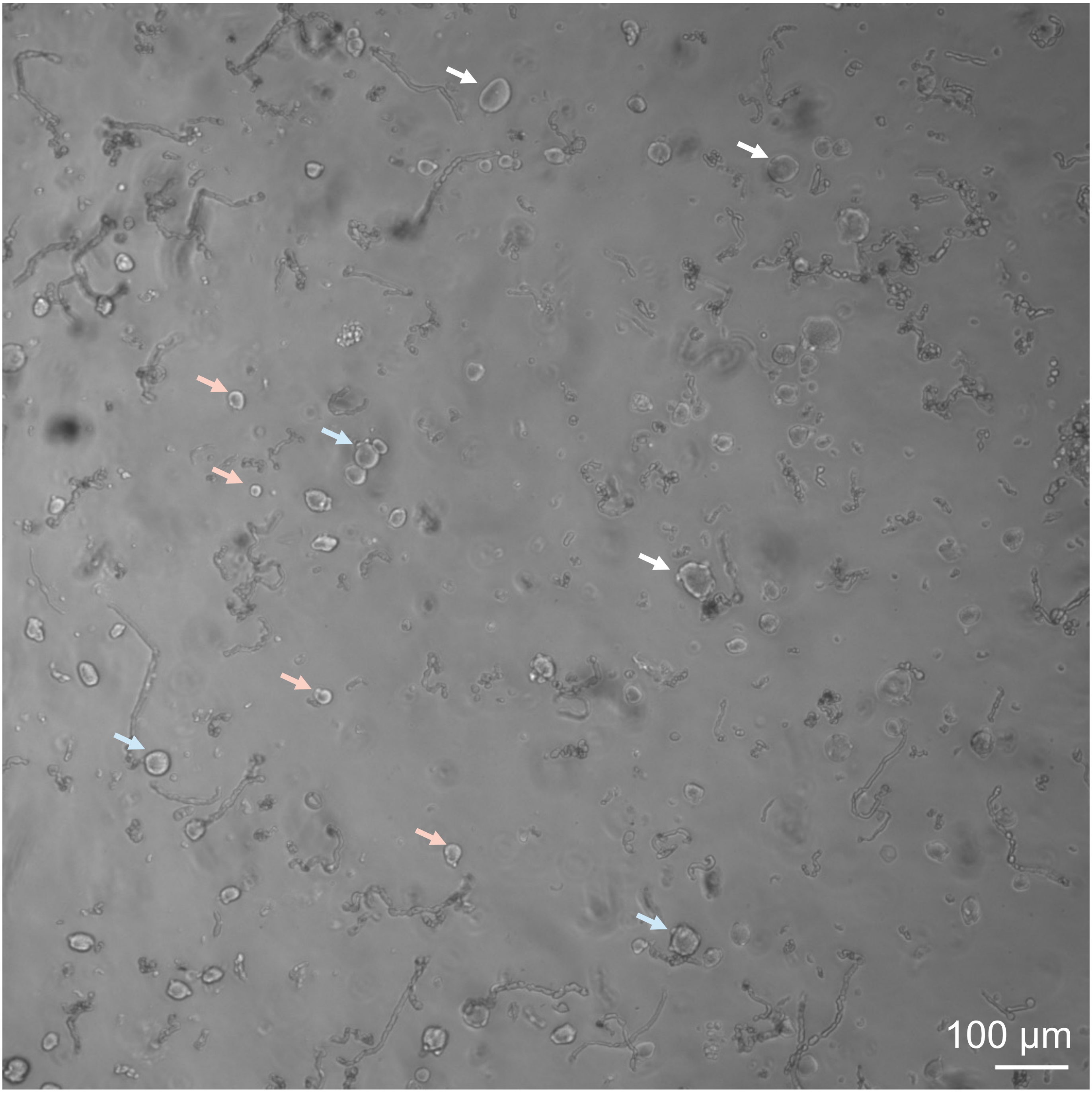

Figure S2. Primary culture of dissociated dorsal root ganglion neurons. The image was taken immediately after dissociation and seeding (0h) into glass bottom dishes. The scale bar indicates 100 µm. Neurons of different sizes can be identified. White arrows indicate examples for large-diameter DRG neurons, blue arrows indicate examples for medium-diameter DRG neurons, and coral arrows indicate examples for small-diameter DRG neurons. The pseudounipolar shape of DRG neurons is lost during the dissociation process, neurons can be identified as round or oval structures with a white corona. In the top left corner, remnants of fibers can be observed. These do not attach to the bottom, and these debris will be removed during medium exchanges. None of the cells have attached yet. Non-neuronal cells cannot easily be identified at this stage.

Figure S3

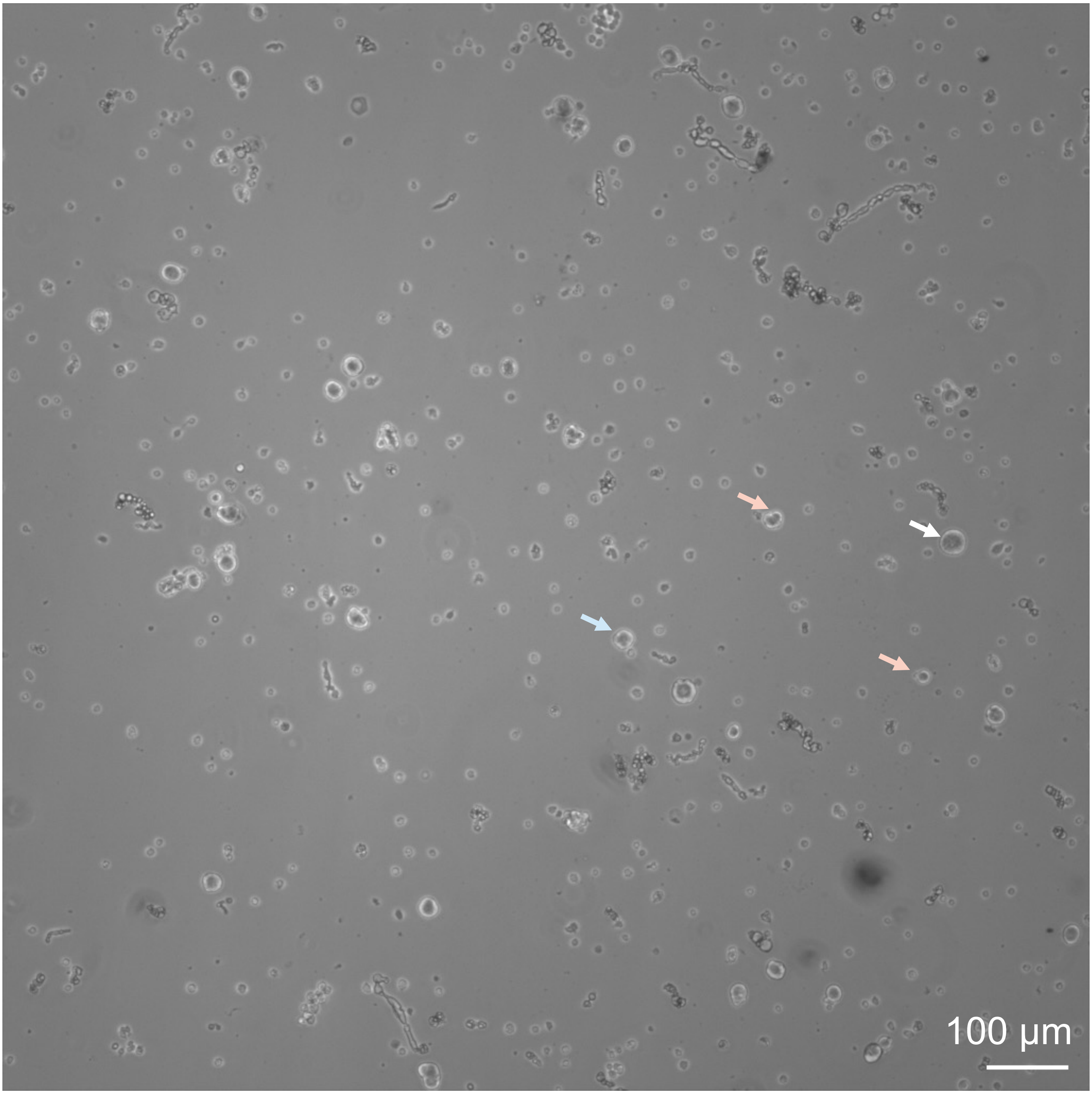

Figure S3. Primary culture of dissociated dorsal root ganglion neurons two hours (2h) after seeding. The image was taken from the same dish as that shown in figure S1. The scale bar indicates 100 µm. DRG neurons of different sizes are difficult to distinguish at this stage. The white arrow indicates a large-diameter DRG neuron. The blue arrow indicates a medium-diameter, and the coral arrows indicate small-diameter DRG neurons. In the top right corner, fibers and debris can be observed. Grey cells without a white corona may be considered non-neuronal cells.

Figure S4

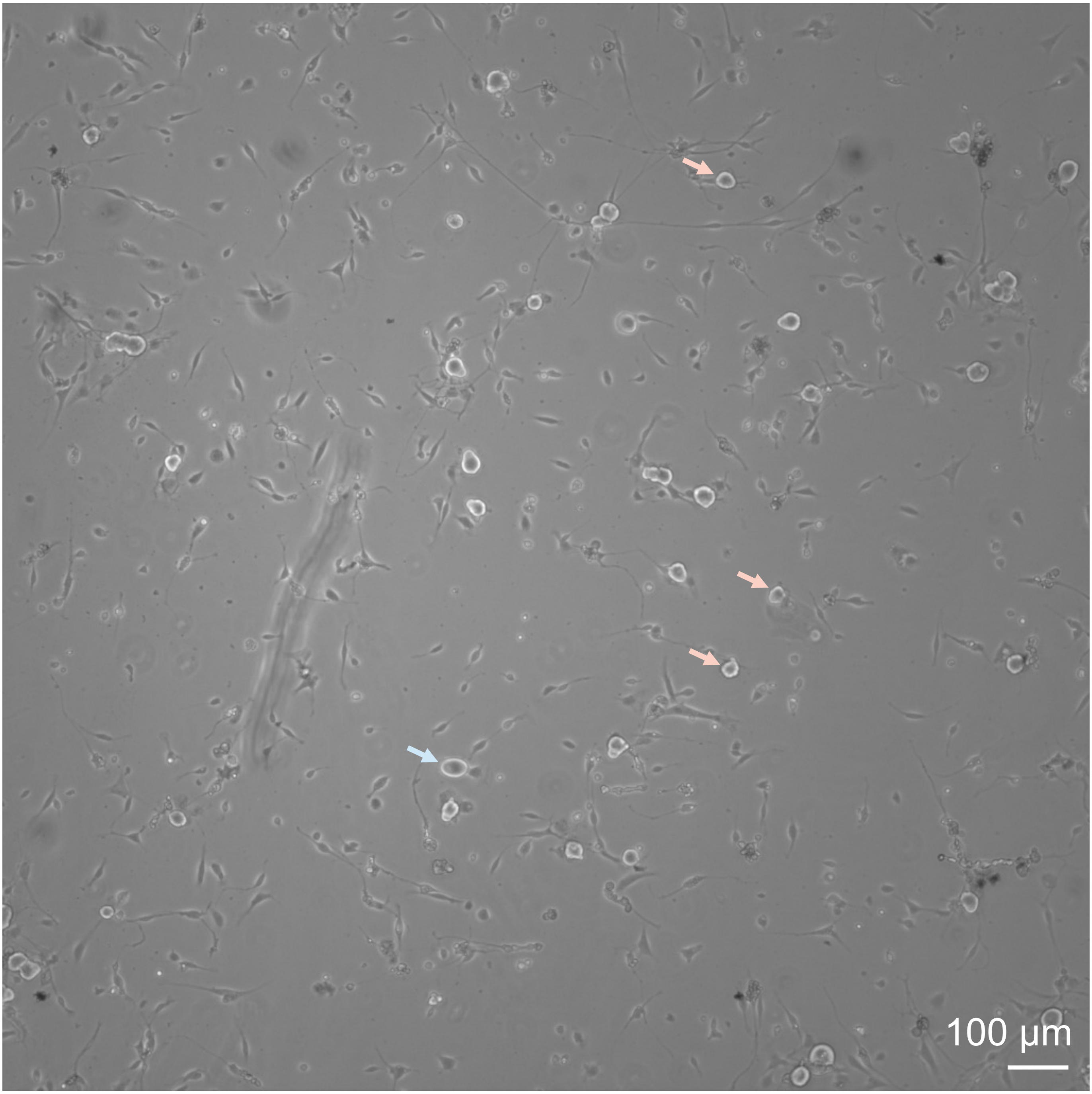

Figure S4. Primary culture of dissociated dorsal root ganglion neurons twenty-four hours (24h) after seeding. The image was taken in the same dish as figures S1 and S2 after the first medium exchange. The culture was treated with ARA-C (1 µM) on the day of seeding. The scale bar indicates 100 µm. No large-diameter neurons can be identified in this field of view. Medium-diameter DRG neurons are indicated by the blue arrow and some small-diameter DRG neurons are indicated by coral arrows. Grey cells without a white corona can be considered non-neuronal cells.

Figure S5

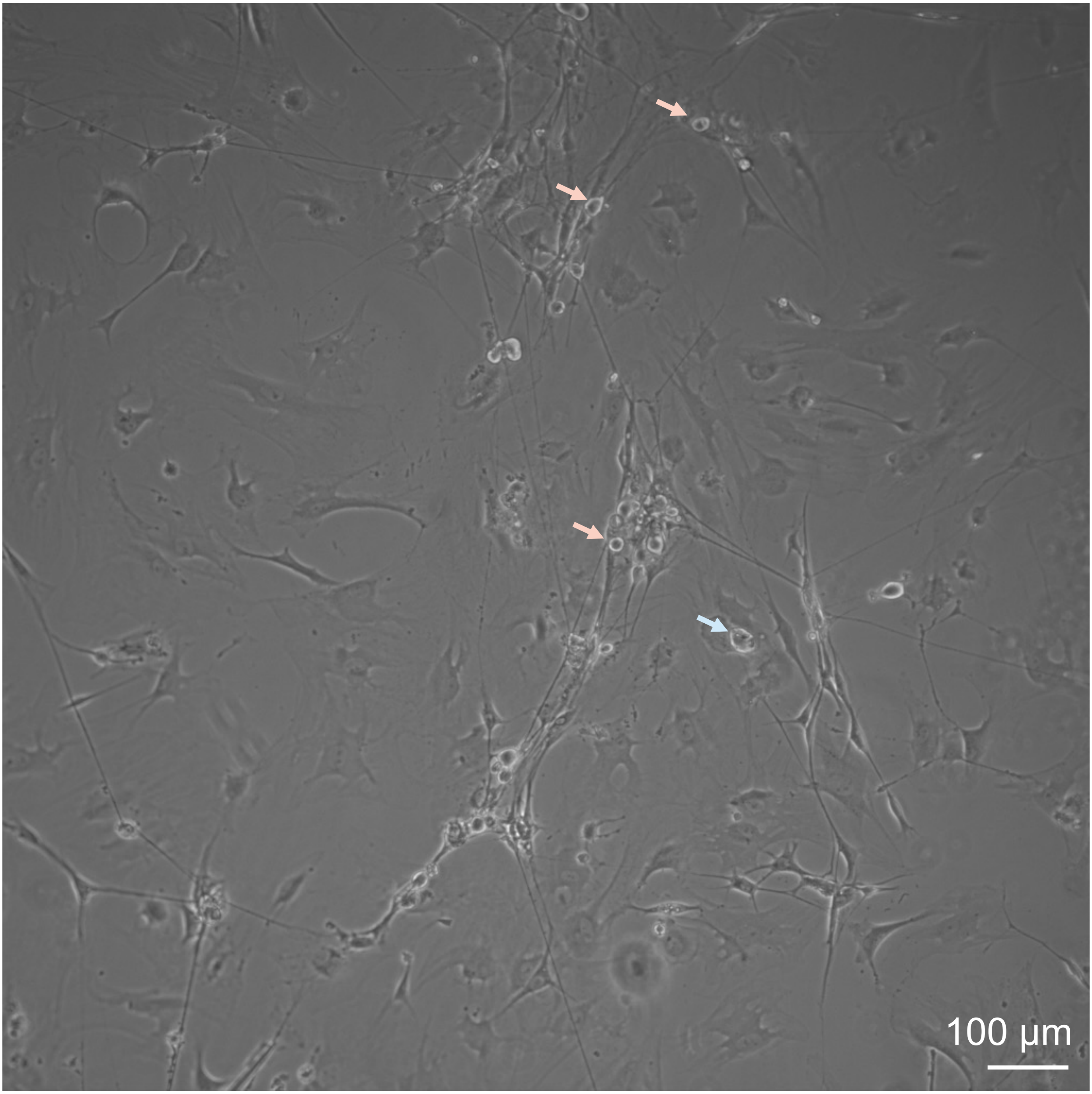

Figure S5. Primary culture of dissociated dorsal root ganglion neurons seven days (7d) after seeding. The image was taken in the same dish as figures S1 and S2. ARA-C (1 µM) was added to the culture was treated with on the day of seeding. The scale bar indicates 100 µm. One medium-sized DRG neuron is indicated by the blue arrow, a few small-diameter DRG neurons are indicated by the coral arrow. Neuronal cells can be identified by their white corona. Grey cells without a white corona can be considered non-neuronal cells. Newly formed connecting fibers between neuronal clusters can be observed.
